# Health literacy measurement: embracing diversity in a strengths-based approach to promote health and equity, and avoid epistemic injustice

**DOI:** 10.1136/bmjgh-2022-009623

**Published:** 2022-09-07

**Authors:** Richard H Osborne, Christina C Cheng, Sandra Nolte, Shandell Elmer, Stephane Besancon, Shyam Sundar Budhathoki, Xavier Debussche, Sónia Dias, Peter Kolarčik, Maria Isabel Loureiro, Helle Maindal, Dulce Nascimento do O, James A Smith, Astrid Wahl, Gerald R Elsworth, Melanie Hawkins

**Affiliations:** 1 Centre for Global Health and Equity, Swinburne University of Technology, Melbourne, Victoria, Australia; 2 Department of Public Health, University of Copenhagen, Kobenhavn, Denmark; 3 Charité Universitätsmedizin, Berlin, Germany; 4 Santé Diabète Headquarter, Grenoble, France; 5 Santé Diabète delegation of Mali, Bamako, Mali; 6 Department of Primary Care and Public Health, School of Public Health, Imperial College London, St. Mary’s Campus, London, UK; 7 Nepalese Society of Community Medicine, Lalitpur, Nepal; 8 Centre Expert Plaies Chroniques, Centre Hospitalier Max Querrien Paimpol, Paimpol, France; 9 NOVA National School of Public Health, Universidade Nova de Lisboa, Lisboa, Portugal; 10 Department of Health Psychology and Research Methodology, Pavol Jozef Šafárik University, Košice, Slovakia; 11 Department of Public Health, Aarhus University, Aarhus, Denmark; 12 Associação Protectora dos Diabéticos, Lisbon, Portugal; 13 College of Medicine and Public Health, Flinders University, Adelaide, South Australia, Australia; 14 Department of Interdisciplinary Health Sciences, University of Oslo, Oslo, Norway

**Keywords:** public health, health policy, community-based survey, health services research, health education and promotion

## Abstract

Definitions of health literacy have evolved from notions of health-related literacy to a multidimensional concept that incorporates the importance of social and cultural knowledge, practices and contexts. This evolution is evident in the development of instruments that seek to measure health literacy in different ways. Health literacy measurement is important for global health because diverse stakeholders, including the WHO, use these data to inform health practice and policy, and to understand sources of inequity. In this Practice paper, we explore the potential for negative consequences, bias and epistemic injustice to occur when health literacy instruments are used across settings without due regard for the lived experiences of people in various contexts from whom data are collected. A health literacy measurement approach that is emic-sensitive, strengths based and solution oriented is needed to minimise biased data interpretation and use and to avoid epistemic injustice.

**Summary box**

- Epistemic injustice is unfair discrimination and exclusion of some groups of people in their capacity as knowers or holders of knowledge, especially groups that are regarded as vulnerable or disadvantaged.
- Measurement validity requires qualitative and quantitative evidence that questionnaire items are understood (and responded to) in the same way across different contexts—such as health systems, entitlements to services and cultural practices—because inaccurate measurement and erroneous score interpretation (including cut-off scores that are not empirically linked to health outcomes or health policy) can lead to unjust and unfair consequences.
- Health literacy is a multidimensional concept and includes measurement of people’s strengths, challenges and preferences as they navigate social experiences for managing health, yet most health literacy measures are developed from Western perspectives of knowing and may not be in harmony with other worldviews.
- To advance global health, we need a health literacy measurement approach that is guided by participatory epistemology and informed by the strengths and locally derived solutions of communities.
- A strengths-based and solution-oriented framework to health literacy measurement that embraces local perspectives, self-determination and strengths will minimise epistemic injustice and provide decision makers with appropriate and meaningful information to promote health and equity.

## Introduction

The concept of health literacy has evolved from health-related literacy to a multidimensional concept that includes social and cultural knowledge, practices and contexts.^1 2^ Initial health literacy measures were English language-based health-related reading and numeracy tests^3 4^ and word recognition tests.^5^ The main purpose of these measures was to inform health information provision to patients. Health literacy then expanded to include health promotion concepts related to an individual’s ability to access, understand, appraise and use health information. A range of instruments were developed to measure some aspects of these attributes, including the European Health Literacy Scale,^6^ which also attempts to compare health literacy across countries. Another recent instrument—the Health Literacy Questionnaire (HLQ)^7^—recognises that health literacy is multidimensional, includes scales to measure social dimensions and people’s lived experiences of engaging with health workers, and was developed to understand patterns of health literacy strengths, needs and preferences in populations. No widely used or accepted health literacy instruments have been developed to account for different cultural and social worldviews or the influences on questionnaire responses that come from the contexts in which people live. Context includes country, culture, language, political influences, type of social and health systems, health conditions, geographical location and other contextual factors that may systemically influence the ways in which a person might choose their answers on a questionnaire. The development of a measurement instrument is necessarily influenced by the context and values of the instrument’s authors and by the measurement and construct definitions on which the instrument is based. To date, health literacy instruments are based on Western definitions of health literacy and measurement, and these may or may not be in harmony with the worldviews of all participants in the studies using these instruments.

The data generated from health literacy instruments are being used to inform health practice and policy. In line with modern measurement theory,^8 9^ the effective use of health literacy data requires evidence that supports the intended interpretation and subsequent use of the data in defined contexts. Context is crucial because there are factors that can influence people’s responses and lead to bias that may result in negative consequences,^8^ such as epistemic injustice.^10 11^ Epistemic injustice is unfair discrimination and exclusion of some groups of people in their capacity as knowers or holders of knowledge.^12^ In health, epistemic injustice occurs when people are ‘regarded as lacking credibility or authority to speak about their experience of their illness or their preferences and interests when making medical decisions’.^13^ An example of this is the use of a survey derived from a worldview that is incompatible with the groups being surveyed; for example, an instrument derived from Western beliefs of individual decision making being applied in contexts of communal decision making or non-Western beliefs, or in mixed or minority populations. To advance global health, we need a measurement approach that is guided by participatory epistemology^14^ and informed by the health literacy strengths and locally derived solutions of communities. Such an approach to measurement is intentionally inclusive of diverse forms and sources of knowledge and means that public health responses are based on local ways of seeing, knowing and experiencing the social environment.

### Understanding measurement

The science behind questionnaire-based measurement started in educational and psychological testing.^8^ In these fields, theories underpinning and processes for determining, valid interpretation of scores were incrementally developed and refined because inaccurate measurement and erroneous score interpretation can lead to unjust and unfair consequences for individuals and institutions.^8 15^ There have been decades of defining and building educational curricula, aligning these curricula to empirically defined testing criteria and evidencing reproducible results across a state or country. Accurate detection and diagnosis of diffuse psychological phenomena may need long questionnaires (eg, the Minnesota Multiphase Personality Inventory),^16^ although decisions about clearly defined, often clinical conditions (eg, depression) may be made using short questionnaires. Confidence in the utility, fairness and equity of cut-off scores for educational and clinical decision making is achieved through longitudinal assessments and evaluations by experts who are guided by clearly defined standards, consensus and decades of professional experience.^15 17^

Over decades, scientists in psychometrics, education and psychology have developed theory and practice to underpin measurement validity testing, resulting in the publication of the authoritative Standards for Psychological and Educational Testing.^8^ Fundamental to modern measurement theory and practice is that validity is understood as the extent to which empirical evidence and theory support score interpretation and use within a measurement context. Validation is a process that ‘…involves accumulating relevant evidence to provide a sound scientific basis for the proposed score interpretations’.^8^ Validity is about the meaning of data in a context for a decision purpose. It is for this reason that it is not a questionnaire that is validated but the inferences and interpretations drawn from data for a specific use within a particular context.^8 9 18^

### The health sector has not kept pace with measurement theory and practice

Patient-reported outcome measures (PROMs) are frequently published and used without the support of robust theory, application of measurement science or substantive evidence that they deliver meaningful data.^19–21^ With readily available statistical software, non-psychometricians who have limited knowledge of modern measurement theory and practice, develop and inappropriately label their questionnaires as ‘validated’. These PROMs are subsequently applied by others in ways and in contexts for which they may not be designed nor intended (eg, in other languages, cultures or diseases). In the area of health literacy, reviews have noted that limited or unsubstantiated evidence is provided to support measurement claims.^21 22^

The claim that a questionnaire is ‘valid’ is problematic because it implies validity is a static property of an instrument and that no further testing is required. Researchers and clinicians who are not familiar with measurement theory then uncritically or naively apply the questionnaire in their own contexts and make claims about what the data mean without evaluating or generating appropriate validity evidence to support their data interpretation and use claims. This can lead to flawed reporting and negative consequences for service users and health policies, which can result in injustices and lead to the widening of health disparities.^11 23^ Developers of early health literacy tests provided high and low cut-off scores based on, for example, data from the USA in the 1990s.^3^ The cut-off points were determined through correlation with a reference sample of adults at two public hospitals and have not been reproduced or updated.^24^ While this cut-off approach allows for ease of use, there is little scientific justification or evidence to determine if a particular level of health literacy puts a person at risk of a poor health event. This approach may provide a dramatic black and white (high/low) result,^25^ but such a result is unverifiable and consequently problematic because it is not evidence based. High/low cut-off scores promote deficit thinking about health literacy, which can be misleading and stigmatising, especially among groups already experiencing vulnerability and disadvantage. To avoid stigmatisation, measurement of health literacy must be considered in context and recognise strengths,^26^ especially as health literacy is a multidimensional concept.^2^ While people may have some health literacy challenges (eg, reading and understanding health information by themselves), they may also have strengths (eg, clinician/family support, understanding how to access health services), which renders the concept of overall high/low health literacy nonsensical.

### Intended purpose of measurement needs to be precise

Standardised health assessments that measure well-defined clinical attributes can be reproducibly applied across groups and cultures. The Global Burden of Disease Initiative is an example where standards for disease classification enable global measurement and comparison between countries. In this initiative, the number of people with specific diseases were counted, and then a disability adjusted life year weight, generated from a global weighting exercise, was applied to the epidemiological findings to generate estimates of burden of disease. When the disease states are concrete and standardised, the measurement of these can be standardised.

In contrast, concepts that are based on experiences or perceptions (eg, health literacy) are more challenging to define and use in surveys. These concepts are called latent constructs and cannot be directly observed or measured. Measurement of latent constructs requires a set of indicators (questionnaire items) that must be equally coherent and meaningful to the full range of potential respondents. A construct derived from Western theory, such as health literacy, may be well articulated for and suited to research by Western researchers and in Western populations but may not be relevant to populations with different worldviews. Consequently, development of a questionnaire must result in each item providing a unique and carefully articulated micronarrative that relates to the lives of all potential respondents and, together, all items represent the intended latent construct.

There are many definitions of health literacy, which are necessary given the diverse contexts (eg, health conditions, health settings) and purposes of measurement (eg, decision making for healthcare, community services, health policy). Development and use of health literacy instruments occur in a variety of ways but should relate to a stated definition.^1 21 22^ A measurement instrument must be an operationalisation of a precise definition because the data generated seek to inform healthcare decisions or policies that usually have implications for people’s health, and can increase or decrease health inequities.^1^ It is incumbent on questionnaire developers to provide evidence that their specific definition is well-founded and has been used in the development of items that do indeed measure the intended construct with precision. Despite well-developed instruments, developers and users need to understand that every measurement process will produce data sets that have error and the potential for bias (ie, threats to data validity), and that these errors and biases can change depending on the context in which data are collected (eg, language, culture, demographic or geographical setting).

### Understanding context and bias

Information derived from health literacy surveys may indicate that differences exist between groups and between countries. These data have the potential to provide insight into individual, social and cultural determinants of health among groups experiencing disadvantage.^27^ Experiences of disadvantage are caused by disparities in the social, economic and environmental structures of societies that lead to inequitiable distribution of resources to some groups of people, including resources that support people to find, understand and use health information and care.^26^ While differences and similarities between groups may reflect true health literacy, data might be biased if certain groups interpret and answer questions differently, not because of their health literacy, but because of factors related to, for example, their age, language or culture, or even the worldview of and values held by the questionnaire developers.^28^

In communal settings, the health literacy of the most influential family member or peer network may be the strongest determinant of an individual’s health literacy.^2 29^ A questionnaire developed from an individualistic perspective might classify an individual from a communal culture as having low health literacy, yet this individual could have good access and many health resources at their fingertips through their community. Most health literacy measures are developed from a Western perspective. Measurement has been central to Western ways of knowing where it is assumed that empirical methods can provide ‘objective’ insights. Problems occur when measures that have been developed by and for Western populations are privileged over other ways of knowing. To apply these measures in non-Western contexts, with no consideration of local emic perspectives and no knowledge input from local people, is an epistemic injustice; an often unintended (or unnoticed) consequence of measurement.^10 12^

In short, for valid comparisons of groups or countries, there must be evidence to show unbiased estimates of group differences (ie, measurement invariance) and that there is an absence of threats to validity, such as construct under-representation (ie, missing fundamental elements of the intended construct) or construct irrelevance (ie, inclusion of elements outside of the intended construct). Failure to adequately demonstrate measurement invariance means that data interpretation could lead to false conclusions about the nature of the health literacy of a group or population, which then can lead to errors in decision making about people’s health and local or national policy. These negative consequences of measurement increase health inequities and are particularly detrimental to people who already experience disadvantage.

### Using health literacy measurement for comparison

Through the Programme for International Student Assessment (PISA) initiative, the Organisation for Economic Co-operation and Development has enabled educational achievements of 15-year-old students to be compared across countries that have similar core educational and testing objectives. The comparisons are possible because of decades of defining and standardising a narrow set of curriculum elements and testing criteria for language, mathematical and science competencies. Given the inherent risks of league tables (discussed below), the PISA team recognises that it is incumbent on them to demonstrate that their instruments are robust in all countries and that scores can be compared across countries.^30 31^ However, it is meaningless to compare wide ranging educational outcomes across countries where there are different educational objectives, functional demands (types of jobs) and standards (eg, university entrance standards). Also, many countries have diverse indigenous and migrant populations that may value and use knowledge in ways that are different from the dominant culture, rendering within and between country or group comparisons misleading.

The World Health Organization’s (WHO) Quality of Life Questionnaire-BREF is frequently used to estimate quality of life.^32^ Attempts to show cross-country measurement invariance—fundamental for country comparisons—have resulted in mixed findings,^33 34^ and this is despite 50 years of research into the quality of life concept. It begs questions about whether concepts like quality of life (and health literacy) can or should be compared across countries and cultures, given the risks of unfair comparisons and negative consequences.

There are important evidence considerations when using a health literacy instrument across countries and cultures.^28^ If the data from the instrument are used to support the design (and implementation) of policies and services within countries, evidence supporting the three aspects of measurement invariance (ie, configural invariance for factor structures, metric invariance for factor loadings and scalar invariance for item intercepts) across groups within each country can often be established. However, when the data are used to make comparisons across countries, for example, for benchmarking or league tables, evidence supporting scalar invariance is necessary but would be very difficult to achieve. Generation of such evidence would require every country to first demonstrate within-country utility and acceptability of the measure, and then to demonstrate, qualitatively and quantitatively, at the item and construct levels, that the questions are understood (and responded to) in the same way, despite different contexts, such as health systems, entitlements to services and cultural practices.

Authentic engagement with and redistribution of power and leadership to people with lived experience enables identification of local health literacy constructs that play a stronger role in determining health and equity outcomes than the hypothesised constructs developed from an etic (outsider) perspective. This approach of co-led or even local-led research requires a commitment to participatory epistemology, where the engagement, input and leadership of local people is an end in itself (ie, a research outcome), and where researcher reflexivity is critical.^14^ Primacy must be given to the local context because without this attention to local emic needs, there is risk of epistemic injustice and harm.

### First do no harm

Application of a health literacy questionnaire to rank and compare groups and countries (ie, league tables) may cause harm (box 1), especially when the measurement instruments are inaccurate for a country context and without applying advanced statistical procedures.^23 35^ With careful planning, harms can be avoided. The development of a universal health literacy questionnaire would require an extensive global ethnographic consultation with well selected and diverse stakeholders within and between countries to avoid data that exhibit top-down paternalistic measurement biases. Enacting participatory epistemology through genuine collaborative and codesign processes—which would take several years—might avoid the development of a questionnaire that omits perspectives of local and regional stakeholders (epistemic injustice) and promulgates health inequities.

Box 1 **Pitfalls and potential harms of cross-country and cross-cultural health literacy assessment**

- Using a definition and assessment instrument grounded in a worldview and culture that is incompatible with non-Western people, for example, an instrument derived from Western beliefs of self-determination and individual decision making applied in contexts of communal and/or non-Western beliefs, or in mixed or minority populations.
- Applying arbitrarily derived cut-off scores—that are not empirically linked to health outcomes or health policy—to label individuals or groups as having adequate or inadequate health literacy.
- Using insufficiently tested measurement instruments that potentially falsely rank a group or country’s position, leading to shaming (low ranking) or complacency (high ranking).
- The exclusion of groups—due to their non-dominant culture, language or other characteristic—in surveys, leading to under representation of potential beneficiaries. This can be due to high burden of measurement and/or low perceived relevance for Indigenous groups, or people with high health literacy needs, people with disability, migrants or language minority groups.

Given the risk of league tables, and that there is already ample evidence of the societal groups that are likely to be classified as having low health literacy, what is needed now is a strengths-based, solution-oriented approach to health literacy measurement to identify clear information about the actions that need to be put in place to improve health outcomes and reduce health disparities.

### A strengths-based, solution-oriented approach to measurement is fundamental

Community development, clinical care and agenda setting have or are moving from approaches that look for deficits (eg, what people or communities can’t do or don’t have, which can lead to victim blaming) to locally led programmes that look for strengths in what people or communities have or can do, and how these can be assets to build on. A deficit approach is characterised by assigning individuals and groups to having insufficient or inadequate levels of an attribute. To advance the field of health literacy, a strengths-based approach to measurement is needed to move beyond deficit-based research and clinical practice that highlight poorer health literacy (and health outcomes) in one individual or group compared with another, and perpetuate deficit narratives that contribute to stigma, stereotypes and marginalisation.^36^

A strengths-based and solution-oriented approach to health literacy measurement maps the assets, challenges and preferences of community members in their contexts, and enables health professionals and services to use these data to inform locally appropriate programmes and policies to improve health literacy responsiveness.^2^ Focusing on strengths does not deny that health inequities exist; rather, such a focus highlights existing individual, health professional, health service, and local and national government capacities to address health issues. Methods where data are generated by local communities for local communities (including communities experiencing disadvantage) are consistent with contemporary indigenous health development models.^26^ The WHO’s influential report Nothing for us, without us^37^ is now considered best practice for community development. Strengths-based measurement is integral to the recommendations in WHO’s report Health Literacy Development for the Prevention and Control of Noncommunicable Diseases.^2^ A health literacy measurement process that embraces the diverse voices of people with lived experience is more likely to minimise epistemic injustices and threats to data validity (ie, bias) and provide information to decision makers that is appropriate, meaningful and useful, especially when positioned in a strengths-based and solution-oriented framework that emphasises people’s self-determination and assets.

The WHO’s 2016 Shanghai Declaration remains a guiding light for health promotion and public health globally.^38^ It draws attention to the critical role of local leadership, especially through municipal leaders. Health literacy measurement that is underpinned by modern measurement theory and practice, that is sensitive to and inclusive of emic perspectives, and that produces strengths-based data will usefully and appropriately guide local health leaders in locally derived solutions for what to do and how to do it to develop health literacy and promote health and equity outcomes.

## Data Availability

No data are available.
